# Supplementary material for: High Glucose Induces in HK2 Kidney Cells an IFN–Dependent ZIKV Antiviral Status Fueled by Viperin
Source: Biomedicines. 2022 Jul 1;10(7):1577. doi: 10.3390/biomedicines10071577 (PMC9313244; doi:10.3390/biomedicines10071577)
Supplement: Supplementary file 1 [file biomedicines-10-01577-s001.zip › biomedicines-1774038-supplementary.pdf]

Supplementary information

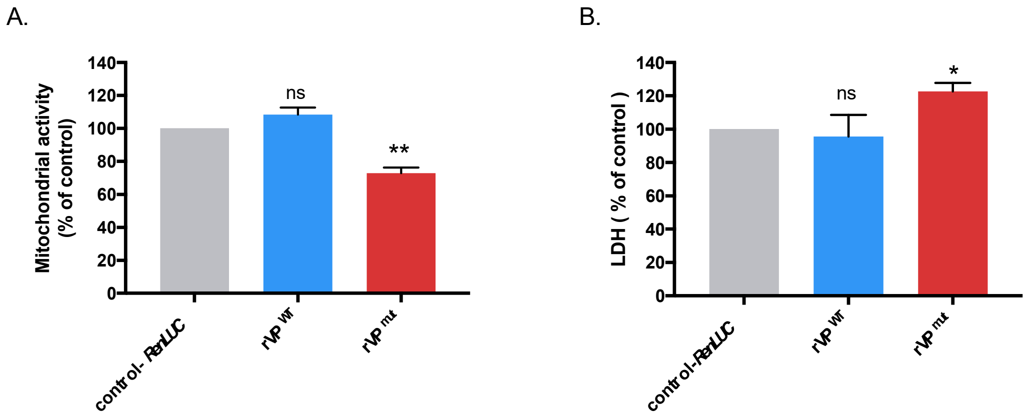

Figure S1. Cytopathic effects due to rVP^{wt} and rVP^{mut} plasmids expression in HK−2 cells. HK−2 cells were transfected with recombinant Viperin wild type (rVP^{wt}) and recombinant Viperin mutant (rVP^{mut}) plasmids for 48 h with a plasmid expressing ren-LUC as a control. (**A**) The metabolic activity of transfected HK−2 cells was determined using 3-[4,5-dimethylthiazol-2-yl]-2,5-diphenyltetrazolium bromide (MTT) assay. (**B**) Quantification of Lactate Dehydrogenase (LDH) release was performed to evaluate the membrane integrity of transfected HK-2 cells. Data were obtained by measuring the absorbance at 570 nm and 490 nm for MTT and LDH assays respectively, with a background subtraction of the absorbance at 690 nm. Results are shown as means ± SD of three independent experiments. Unpaired t-test demonstrates that differences are either non-significant (ns) or statistically significant (* $p < 0.05$ and ** $p < 0.01$).

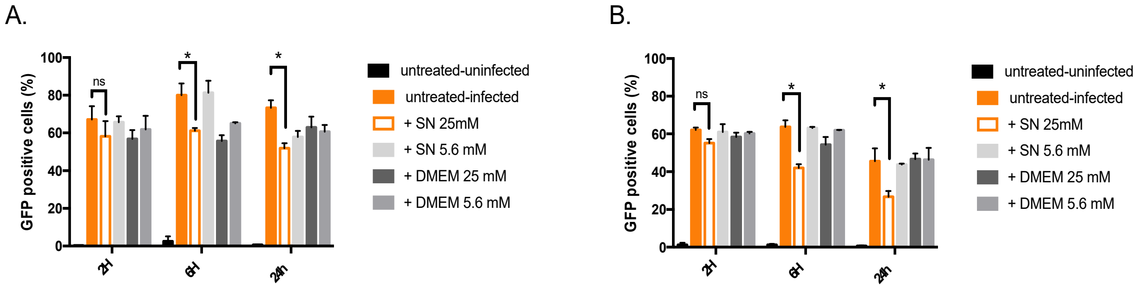

Figure S2. Effect of high-glucose HK−2 cells supernatant on ZIKV infection. The supernatant of HK−2 cells grown in high-glucose condition for a 24 h (**A**) or 48 h (**B**) period was transferred on naive HK−2 cells under low-glucose condition at 2 h, 6 h and 24 h pre-infection. The cells were infected with ZIKV^{GFP} at a MOI of 1 for 24 h. The cells were then fixed and subjected to flow cytometry analysis. Results are shown as means ± SD of three independent experiments. Two-way ANOVA shows that differences are either non-significant (ns) or significant (* $p < 0.05$).

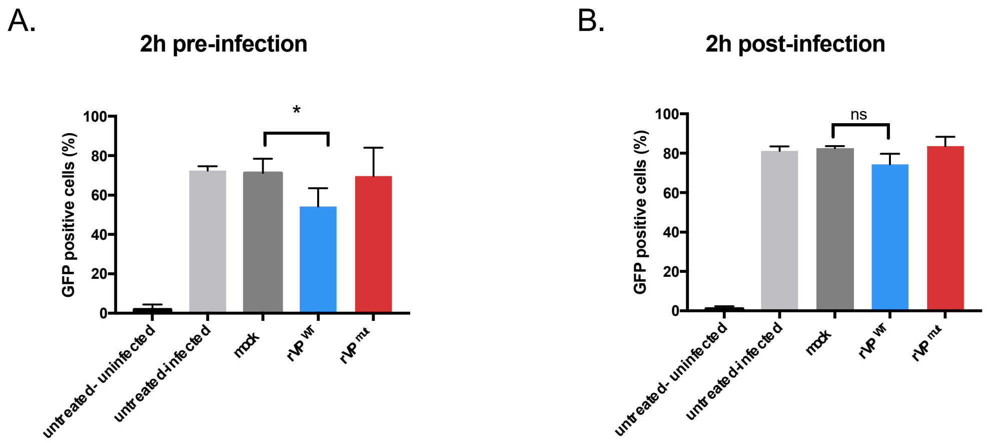

Figure S3. Paracrine effect of viperin−HK-2 transfected cells on ZIKV growth in A549 human cells. HK−2 cells were transfected with recombinant Viperin wild type (rVP^{wt}) and recombinant Viperin mutant (rVP^{mut}) plasmids for 24 h. The supernatant of transfected cells was collected and transferred on A549 cells at (**A**) 2 h pre-infection and (**B**) 2 h post-infection. The cells were infected for 24 h with ZIKV^{GFP} at a MOI of 1. Results are presented as means ± SD of three independent experiments. Unpaired t-test shows that differences are either non-significant (ns) or significant (* $p < 0.05$).
